# From macrophage biology to macrophage-based cellular immunotherapies

**DOI:** 10.1038/s41434-021-00221-5

**Published:** 2021-02-04

**Authors:** Elvira Mass, Nico Lachmann

**Affiliations:** 1 Developmental Biology of the Immune System, Life & Medical Sciences (LIMES) Institute, University of Bonn, Bonn, Germany; 2 Translational Hematology of Congenital Diseases, Institute of Experimental Hematology, Hannover Medical School, Hannover, Germany; 3 REBIRTH Research Center for Translational and Regenerative Medicine, Hannover, Germany; 4 Department for Pediatric Pulmonology, Allergology and Neonatology, Hannover Medical School, Hannover, Germany

**Keywords:** Immunotherapy, Innate immune cells

## The concept of macrophages

**Fig. 1:**
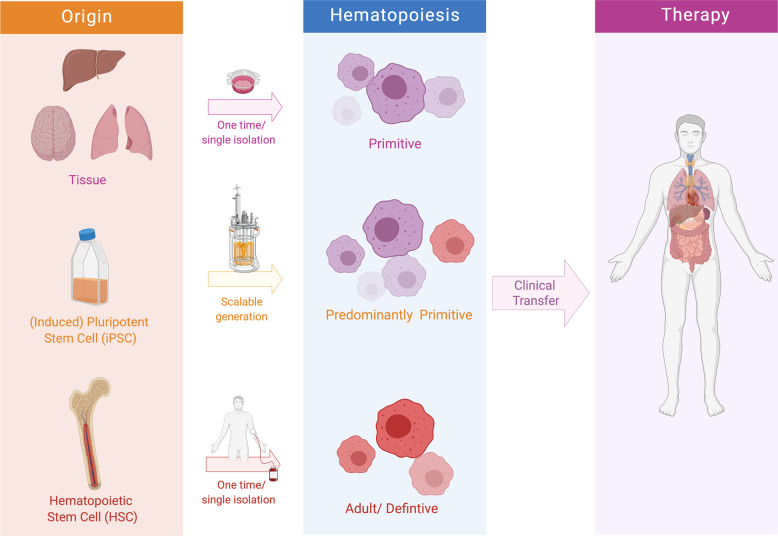
Subsets of macrophages for adoptive cell therapies. Macrophages may be isolated or generated from tissues, induced pluripotent stem cells (iPSCs), or hematopoietic stem cells (HSCs). In fact, the origin and the starting cell material for the derivation of macrophages should be evaluated prior to generation. While the iPSC technology would allow for scalable and continuous generation of predominantly primitive macrophages, generation of HSC- or peripheral blood derived monocyte-derived macrophages would allow only for a single derivation of definitive cells. Following an, e.g., macrophage enhancement and/or purification step, generated macrophages may be infused into the patient to improve disease symptoms.

As far back as 1882, Ilya (Elie) Metchnikoff was the first to discover macrophages (MΦ) and their important role in host defense [1]. Now, almost 140 years later, MΦ are known as incredible cell types with unique functions and capabilities. In fact, Ilya Metchnikoff paved our understanding of peripheral blood monocytes and MΦ in the clearance of pathogens, while follow-up studies described MΦ in various organs in which their defined functions are directly linked to a divergent onset (Fig. 1).

MΦ are found in all organs, typically forming a three-dimensional network that supports tissue function by production of growth factors and phagocytosis of dead and dying cells during steady state. Moreover, MΦ are among the first cells encountering pathogens, debris, or tumor cells in diseased tissue. Given these and other multifarious functions and their important role in the onset and/or progression of various diseases, MΦ are therefore rendered as a perfect candidate in cell-based immunotherapies.

Against a great body of newly emerging data, scientists still encounter in the MΦ research literature the terminology of MΦ polarization, most commonly into the M1 (classically activated) and M2 (alternatively activated) phenotypes (see also review [2]). Thus, also therapy approaches aim at using this apparent plasticity of MΦ and “simply” try to switch them from M1 to M2 or the other way around, depending on whether anti- or pro-inflammatory responses are required. However, these M1/M2 definitions stem from cells treated with interferon-γ or interleukin-4, respectively, and ignore the fact that these defined and isolated stimuli in vitro do not emerge in vivo. In contrast, under physiological conditions a plethora of stimuli (e.g., cytokines, bacteria, foreign antigens, or the tissue niche) modulate MΦ function in an orchestrated fashion, which can be difficult to recapitulate in vitro [2].

## Macrophage ontogeny = macrophage function?

Given the demonstration in recent years that tissue-resident and recruited MΦ represent different developmental lineages, there is growing evidence for their nonredundant functions during steady state and disease. While most tissues harbor only a minor fraction of monocyte-derived MΦ, the majority of tissue-resident MΦ originate from erythro-myeloid progenitors (EMPs) in the yolk sac [3] that are maintained throughout life largely independent of the circulating hematopoietic system by local proliferation. Thus, the observed plasticity and multi-functionality of MΦ in healthy and diseased tissues may not only be triggered by environmental cues, but also already determined by MΦ ontogeny. One such example is the liver: EMP-derived Kupffer cells emerge early during embryogenesis and are involved in hepatocyte metabolism and erythrocyte development and recycling—just to name a few of many functions—while monocyte-derived liver capsular MΦ sense and control entrance of peritoneal bacteria via recruitment of neutrophils [4].

That origin matters for MΦ identity and function has been shown in studies in which an empty MΦ niche was replenished by transplanted MΦ progenitors of fetal or adult origin or by already differentiated MΦ from other tissues. While this concept would be applicable for, e.g., congenital diseases (e.g., CSF2RA/B deficiency, see below), other adoptive transfer scenarios would rely on the depletion of the endogenous MΦ pool to allow homing of cells. Clodronate liposomes, CSF1R antagonists, or sophisticated irradiation schedules may be applied alone or in combination to sufficiently deplete resident MΦ and/or infiltrating endogenous monocytes, while carefully evaluating potential off-target effects.

Most progenitors and fetal MΦ are highly adaptive to their environment and establish a microglia-like phenotype in the brain or alveolar MΦ (AM) phenotype in the lung, and may even take over certain functions of the original MΦ population. Yet, in some cases MΦ of inappropriate origin show a distinct transcriptional profile and express disease-associated genes [5], or display decreased function during steady state and fail to protect from infection [6].

However, a generalized oversimplification of EMP-derived MΦ being pro-regenerative and anti-inflammatory, while monocyte-derived MΦ are rather pro-inflammatory, does not always apply. Depending on the molecular make-up of the cell or disease progression, EMP-derived MΦ can become pro-inflammatory and drivers of pathophysiology. Mutated EMP-derived microglia can cause neurodegeneration [7], and Kupffer cells experiencing a nonalcoholic steatohepatitis diet for 30 weeks lose their identity and transit toward a pathogenic phenotype similar to monocyte-derived MΦ [8].

MΦ-based therapies should therefore consider the distinct ontogeny and the potential of MΦ and MΦ progenitors to adapt to (one of) these cells. Here, the developmental dichotomy could be represented by induced pluripotent stem cells (iPSCs)- or monocyte-derived MΦ, respectively: while iPSC-derived MΦ (iPSC-MΦ) may be easily harvested and collected for transplantation to mimic EMP-derived tissue-resident MΦ [9], monocytes enriched from the blood of patients would differentiate into pro-inflammatory MΦ. In the future, it may be useful to employ both strategies simultaneously or even to combine this cell-therapy approach with a genetic therapy where one or both macrophage lineages are genetically corrected or modulated to treat diseases since the correct balance of MΦ of distinct origins is what every tissue needs for its homeostasis.

## (Pre)Clinical attempts of macrophage-based therapies

As one attractive clinical roadmap for a MΦ therapy, liver cirrhosis has been introduced by the team of Stuart Forbes, using peripheral blood monocyte-derived MΦ [10]. In a phase I clinical trial (ISRCTN 10368050), the authors isolated monocytes from individuals and infused M-CSF-differentiated MΦ intravenously back into the patients [11]. Designed as a dose-escalation study, the authors could not observe any reaction to the transfusion, neither dose-limiting toxicity nor any sign of macrophage activation syndrome. While no adverse events could be monitored, overall efficacy still needs to be shown in patients. A similar liver-directed approach using MΦ has been applied in the context of the rare disease heme oxygenase-1 (HMOX1) deficiency. In a preclinical study, the infusion of WT MΦ into *Hmox1*-deficient mice could proof engraftment and proliferation of cells and a correction of the disease phenotype [12]. To expand the field of MΦ-based therapies, adoptive transfer of MΦ directly into the lungs has been also recently highlighted for various diseases. In the context of *Csf2r*-deficient pulmonary alveolar proteinosis (PAP), first studies concentrated on bone marrow (BM)-derived MΦ, which have been transferred as a single dose directly into different mouse models, which faithfully recapitulate the clinical phenotype of PAP [13, 14]. Given the longevity and the adaption of transferred BM-MΦ toward an AM phenotype, current attempts are underway to establish an autologous MΦ-cell product, which is genetically corrected and ready to be transferred into CSF2RA-deficient patients. Similar to the approach of BM-derived MΦ, iPSC-MΦ could also be shown therapeutically effective following intrapulmonary transfer into mouse models of PAP or severe combined immunodeficiency. Moreover, the new technique also paved the way for the exploration of iPSC-MΦ in the context of infectious diseases, in which iPSC-MΦ have been used as an antibiotic independent immunotherapeutic approach targeting bacterial infections of the lung and other organs. Beyond these studies, populations of MΦ have been also introduced in the brain (microglia) or bone (osteoclasts) and the impact of these two MΦ-populations in the onset and progression of different diseases has been highlighted. For the latter, a recent study could not only proof the onset of osteoclasts from embryonic EMPs, but could also demonstrate that transfusion of monocytic cells can rescue mice from an adult-onset osteopetrotic phenotype [15]. While microglia also emerge from an early EMP during embryogenesis, human iPSC-MΦ have been shown to functionally integrate into xenografted mouse brain and retain human microglia identity [16]. This observation may extend recent attempts, which directly transfer hematopoietic progenitors directly into the brain. Given the various attempts to adoptively transfer MΦ into various tissues, clearly the fields of organ transplantation and cancer immunotherapy have been targeting MΦ directly or indirectly. While the onset of tumor-associated macrophages is diverse, redirecting MΦ to specifically recognize the tumor by chimeric antigen receptor (CAR) technology has been introduced as a completely new field in MΦ-directed therapies. Redirecting MΦ either by the forced expression of an anti-CD19 or anti-HER2 CAR against leukemia or solid tumors, respectively, and the introduction of an intracellular phagocytosis signaling cascade could impressively demonstrate the future potential of these MΦ [17, 18]. Of note, MΦ have been further manipulated by, e.g., cell intrinsic or extrinsic modifiers and used in different applications such as cancer immunotherapy, renal transplantation, type 1 diabetes, and others [19–21].

Clearly, the seminal potential and function of MΦ and the recent possibilities to generate MΦ in a scalable bioreactor differentiation system pave the way for innovative immunotherapies (Fig. 1). Besides the costly manufacturing, it is crucial to properly evaluate safety-related issues of either autologous or allogenic MΦ in appropriate (pre)clinical studies in order to promote the use of these cells in the clinic to benefit a greater number of patients. Taken together, recent and future insights into the biology and onset of MΦ could further extend our understanding on the therapeutic use of these cells in a variety of different diseases and medical indications (Fig. 1).
